# The NEST Dry-Run Mode: Efficient Dynamic Analysis of Neuronal Network Simulation Code

**DOI:** 10.3389/fninf.2017.00040

**Published:** 2017-06-28

**Authors:** Susanne Kunkel, Wolfram Schenck

**Affiliations:** ^1^Simulation Laboratory Neuroscience, Bernstein Facility for Simulation and Database Technology, Institute for Advanced Simulation, Jülich Aachen Research Alliance, Forschungszentrum JülichJülich, Germany; ^2^Department of Computational Science and Technology, School of Computer Science and Communication, KTH Royal Institute of TechnologyStockholm, Sweden; ^3^Faculty of Engineering and Mathematics, Bielefeld University of Applied SciencesBielefeld, Germany

**Keywords:** profiling, performance analysis, memory footprint, high-performance computing, supercomputer, large-scale simulation, spiking neuronal networks

## Abstract

NEST is a simulator for spiking neuronal networks that commits to a general purpose approach: It allows for high flexibility in the design of network models, and its applications range from small-scale simulations on laptops to brain-scale simulations on supercomputers. Hence, developers need to test their code for various use cases and ensure that changes to code do not impair scalability. However, running a full set of benchmarks on a supercomputer takes up precious compute-time resources and can entail long queuing times. Here, we present the NEST dry-run mode, which enables comprehensive dynamic code analysis without requiring access to high-performance computing facilities. A dry-run simulation is carried out by a single process, which performs all simulation steps except communication as if it was part of a parallel environment with many processes. We show that measurements of memory usage and runtime of neuronal network simulations closely match the corresponding dry-run data. Furthermore, we demonstrate the successful application of the dry-run mode in the areas of profiling and performance modeling.

## 1. Introduction

The neuronal network simulator NEST (Gewaltig and Diesmann, 2007) has an active and growing community of developers and users. Advances in computer hardware on the one hand and the requirements of novel computational models on the other hand push the development of new simulation technology and have made NEST a tool for a broad spectrum of applications. It enables simulations of spiking neuronal networks that differ greatly in size and complexity, and depending on the requirements of the models researchers can run the simulations on their laptops or they can make use of high-performance computing (HPC) facilities.

A simulation with NEST consists of two main phases. During the *build phase* (or *setup phase*) NEST creates neuron and synapse objects and the data structures to access these objects. Afterwards the actual *simulation phase* starts, in which the *main simulation loop* is repeated iteratively. Each iteration comprises the update of synapses and neurons, the exchange of recent spikes between MPI ranks and the delivery of these spikes to their local targets. A description of the fundamental data structures and the main simulation loop of NEST follows in Section 2.1.

The software-development framework around NEST is becoming ever more comprehensive. A testsuite (Eppler et al., 2009b) and continuous integration technology (Zaytsev and Morrison, 2012) help to ensure code quality; models of memory usage (Kunkel et al., 2012) and runtime (Adinets et al., 2015; Schenck et al., 2014) enable the structured analysis of existing code and the design of new, more scalable data structures and algorithms. However, in order to confirm the model predictions and to check for errors that occur only in the supercomputing regime, NEST developers still need to carry out actual simulations on supercomputers that may employ hundred thousands of MPI processes. Running such tests and benchmarks takes up compute-time resources and slows down the code-development process due to long queuing times.

In this manuscript, we present a method, where only one MPI process carries out its part of a distributed simulation with NEST. In the build phase, the process sets up all local data structures as if it took part in a parallel simulation with many processes, and in the simulation phase, it creates *fake spike*s to represent input from other MPI ranks. We refer to this method as the *dry-run mode* of NEST and to the actual distributed neuronal network simulation that corresponds to a specific *dry run* as *real run*. Furthermore, we distinguish between *static* and *dynamic* dry-run mode. In the static mode, the MPI process creates fake spikes according to a predefined firing rate while in the dynamic mode the process uses the spikes of its local neurons to invent the fake remote spikes. In Section 2.2, we provide details on the implementation of the dry-run mode and also an example script that shows how the mode can be enabled.

In computer science, the term “dry run” often refers to hardware tests under controlled conditions, but it is also used in the context of software development to refer to the static analysis of an algorithm by mentally evaluating each step. The NEST dry-run mode enables dynamic code analysis and hence differs from the latter definition. A NEST dry run corresponds more to a hardware dry run: Simulation code is executed in the controlled environment of a single MPI process such that failure cannot cause any severe damage as the simulation does not use any allocated HPC resources.

The definition of the term “dry run” is rather vague as the concept is rather uncommon. Examples of programs that provide a dry-run option are among others the rsync utility (Tridgell, 1999) and the GPAW project (Enkovaara et al., 2010). The command-line tool rsync enables file synchronization and transfer; a dry run produces an output of the potential changes, which the user can check before performing a real run. GPAW enables electronic structure calculations; a dry run allows the user to estimate the memory usage and to inspect the parallelization settings for a given number of MPI processes. In this way, the dry-run mode of GPAW is conceptually similar to the dry-run mode of NEST concerning the build phase. However, we are not aware of any simulation software which is able to perform a dry run of the simulation dynamics.

The dry-run mode of NEST allows developers to investigate the performance of different parts of the code without consuming precious computing time. This requires, however, that dry run and real run exhibit similar memory-access patterns, which may seem questionable given that in dry-run mode most spikes do not originate from real network dynamics. Hence, in Section 3.1 we compare spiking activity, memory usage, and runtime of dry-run and real-run simulations for different network sizes and number of processes.

Within the software-development framework of NEST, the dry-run mode complements the previously developed models of memory usage and runtime as it is an economical way to obtain a realistic performance estimate of a distributed simulation. Kunkel et al. (2012) used a preliminary version of the dry-run mode to verify the predictions of the memory-usage model and in Helias et al. (2012) and Kunkel et al. (2014) the network sizes for the maximum-filling benchmarks were determined using the preliminary dry-run mode, which saved a lot of time and HPC resources. Dry-run simulations are also compatible with established profiling tools (see e.g., Schenck et al., 2014) because it is one and the same NEST binary which is used for conventional NEST operation and for dry runs. Besides, because the dry-run mode only requires a single compute node, developers can debug their NEST code for different regimes of number of processes without requiring access to HPC facilities—a single workstation is sufficient. Dry-run simulations can also help NEST users to estimate the HPC resources that they need to request for planned simulations. In order to demonstrate the usefulness of the dry-run mode we give several sample use cases in Section 3.2.

The conceptual and algorithmic work described here is a module in our long-term collaborative project to provide the technology for neural systems simulations (http://www.nest-initiative.org). We devised the dry-run mode as a software-development method for NEST. However, the concepts that we present here are transferable to other simulators.

## 2. Materials and methods

### 2.1. Neuronal simulator NEST

NEST is a simulation software for spiking neuronal networks of single- and few-compartment neuron models (Gewaltig and Diesmann, 2007; Kunkel et al., 2017). The simulator supports a hybrid parallelization strategy with at least one MPI process per compute node and multi-threading based on OpenMP within each process (Eppler et al., 2007; Plesser et al., 2007). Simulations with NEST can be controlled through the built-in scripting facility SLI or the Python-based user interface PyNEST (Eppler et al., 2009a; Zaytsev and Morrison, 2014).

The development of NEST is coordinated by the NEST Initiative (http://www.nest-initiative.org). NEST is under the GNU General Public License. It can be cloned or forked on GitHub (https://github.com/nest/nest-simulator). Documentation, examples, and releases are available on the website of the NEST Simulator (http://www.nest-simulator.org).

**Figure 1:**
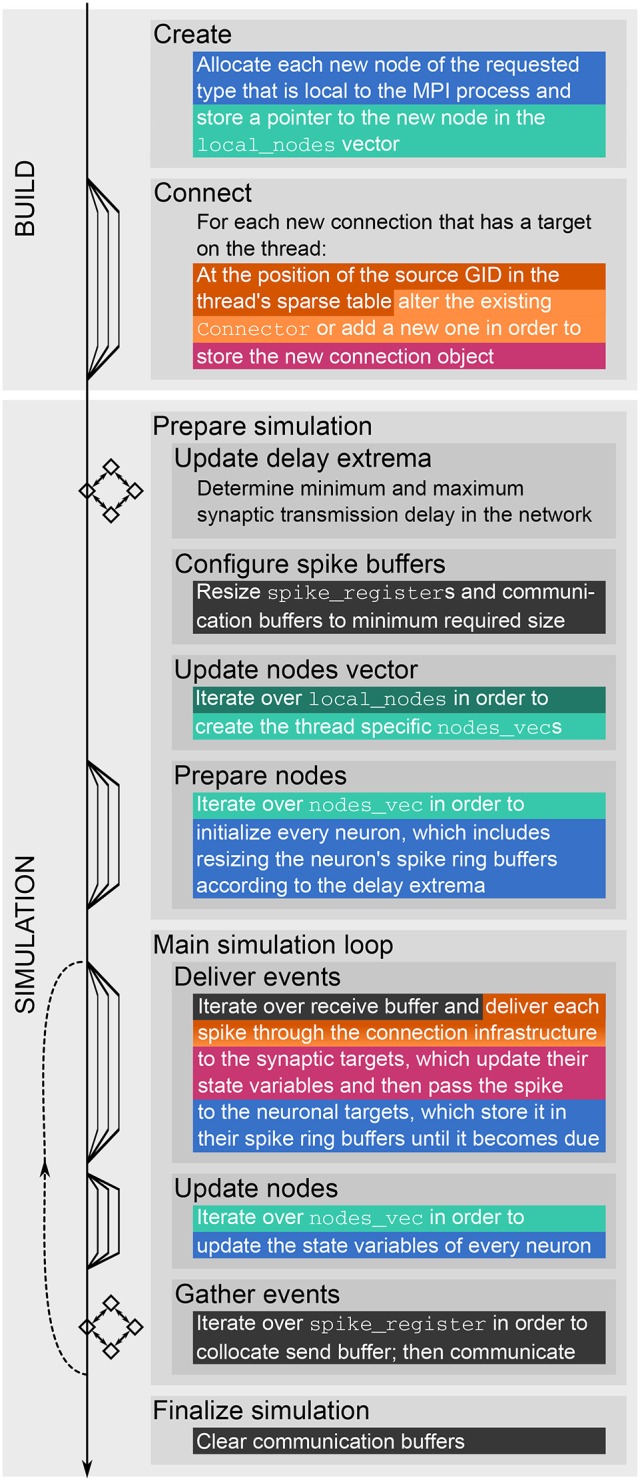
Major steps of build and simulation phase of NEST. The vertical black arrow on the left indicates single-threading and multi-threading (single and multiple lines, respectively), MPI communication (squares with bidirectional arrows), and the repetition of the main simulation cycle (dashed upward pointing arrow). Colored and dark gray text highlighting corresponds to the data structures shown in Figures 2, 3, respectively; they indicate when the data structures are created, changed or accessed.

**Figure 2:**
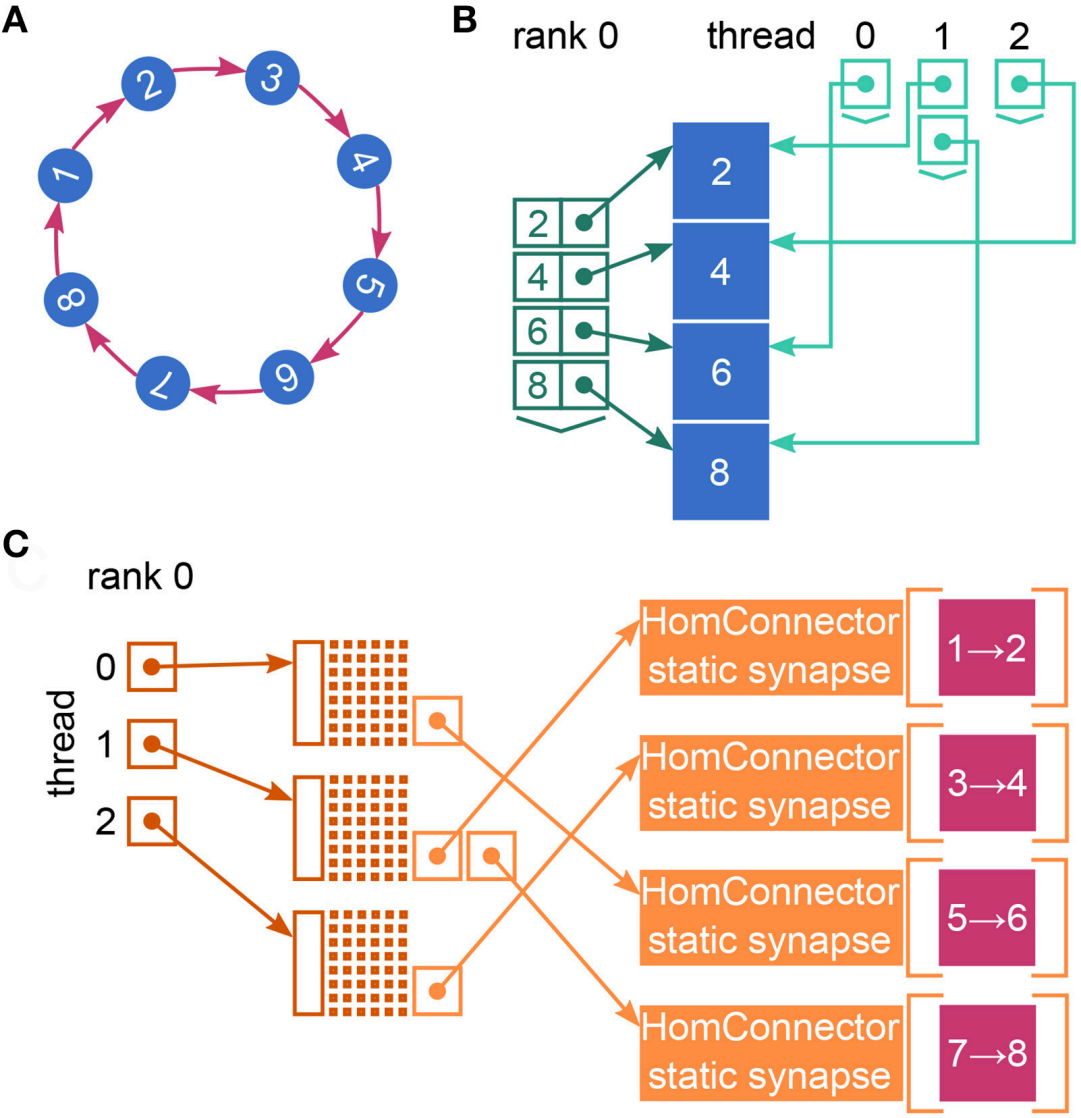
Fundamental data structures in NEST. Data structures on MPI rank 0 for **(A)** an example network of eight neurons with ring-like connectivity, which is simulated using two MPI processes and three threads per process. For simplicity, devices are omitted. **(B)** Neuron infrastructure. For each local neuron (blue squares) the SparseNodeArray local_nodes (dark green) stores a struct of a pointer to the neuron and the neuron's GID. The two-dimensional vector nodes_vec (light green) stores a pointer to the local neurons sorted by thread. **(C)** Connection infrastructure. Each synapse is represented on the thread of its target neuron. Each thread owns a sparsetable (dark orange), which stores a pointer to a Connector (orange) for every source neuron that has targets on the thread. The Connectors hold the local synapses (pink) sorted by type (here only one static_synapse per Connector).

NEST creates most of the required data structures during the *build phase* (or *setup phase*), where each MPI rank operates independently of the other ranks (see Figure 1). Nodes are created and connected according to the user-specified simulation script and each node is assigned a global identifier (*GID*). Typically, nodes are neurons but they can also be devices such as spike detectors. NEST distributes neurons across MPI ranks and OpenMP threads in a round-robin fashion according to their GIDs, which balances the computational load. Devices, however, are duplicated on each thread. Synapses are represented on the same thread as their post-synaptic neurons. Moreover, each thread owns data structures that enable efficient access to its local neurons and synapses (see Figure 2).

**Figure 3:**
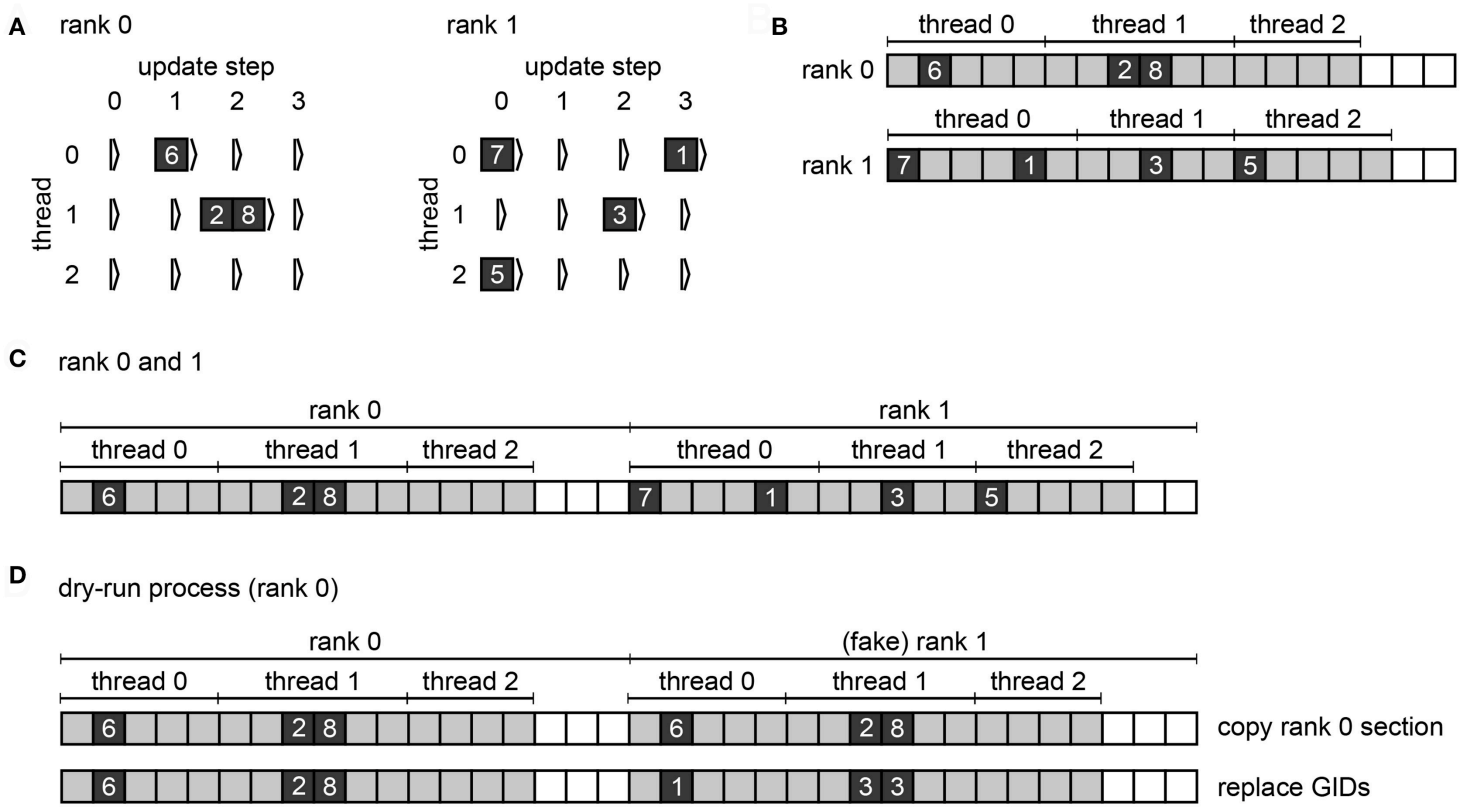
Spike buffers in NEST. Example spike buffers for a simulation cycle with four neuronal update steps and for a network of eight neurons, which is simulated using two MPI processes and three threads per process. **(A)** During neuronal updates the three-dimensional vector spike_register stores the GIDs of the local neurons that spike (dark gray squares) sorted by thread and update step. **(B)** Before MPI communication each rank collocates its send buffer based on the entries in its spike_register. Communication markers (light gray squares) define update step and thread. Buffers may not be completely filled (white squares). **(C)** After MPI communication using MPI_Allgather each rank holds the complete spike data in its receive buffer (global spike buffer), which is the concatenation of the send buffers of all ranks. **(D)** Filling of the global spike buffer in dynamic dry-run mode. The section that belongs to MPI rank 0 is copied to the section that belongs to fake rank 1. The GIDs in the section of rank 1 are then replaced with randomly chosen GIDs of neurons that would be local on rank 1 in the corresponding real-run simulation; the assignment of local neurons to threads 0, 1, and 2 is also respected.

The call to Simulate in the user script triggers the actual *simulation phase*. NEST first prepares the data structures for the simulation and then enters the *simulation loop*, which iterates through *simulation cycle*s until the requested simulation time elapses (see Figure 1). Every simulation cycle starts with the *delivery* of the spikes that occurred during the last cycle. Each thread reads the *global spike buffer* (see Figure 3C) and directs the relevant spikes to the local target synapses, which pass them on to the local target neurons. The delivery step comprises also the update of plastic synapses.

The second step in every simulation cycle is the threaded *update* of all neurons and devices. When an update is triggered, neurons typically propagate their dynamics in submillisecond steps according to the globally defined simulation resolution; we refer to these integration steps as *h-step*s. Thread-specific *spike register*s (see Figure 3A) collect all spikes that the local neurons emit during the current update sorted by h-step.

Every simulation cycle terminates with a *gather* step, which is the communication of the newly generated spikes using MPI_Allgather. To this end spikes need to be transferred from the spike registers to the local MPI *send buffer*s (see Figure 3B), where markers indicate the sections for different h-steps and threads. The send buffers are equally sized on all ranks; their size depends on the maximum number of spikes per rank which was emitted so far during the course of the simulation within one simulation cycle. After the communication, each rank holds the recent spikes from all ranks in its MPI receive buffer, which is the global spike buffer that is processed in the next delivery step.

The minimum synaptic transmission delay in the network defines the interval at which MPI processes need to exchange spikes in order to maintain causality (Morrison and Diesmann, 2008). Hence, the *min-delay interval* or *communication interval* defines also the time interval of the simulation cycle.

### 2.2. Implementation of the dry-run mode

In dry-run mode, NEST is executed only by MPI rank 0 but this rank behaves as if it was part of a distributed simulation with many MPI processes; we refer to all MPI ranks except rank 0 as *fake rank*s as they exist only conceptually but are not instantiated. In the build phase the dry-run process needs to take into account the total number of MPI processes when creating the local data structures, and in the simulation phase the dry-run process needs to generate fake spikes that represent the input from the fake ranks. We describe this in more detail in the following Sections 2.2.1, 2.2.2, respectively.

In order to implement the dry-run mode we used the NEST-kernel parameter num_processes, which is initialized to MPI_Comm_size and then used as reference for the total number of MPI processes throughout the simulation. Typically, NEST users can only inspect num_processes but they cannot manipulate it. In dry-run mode, however, the parameter is unlocked and can be set to the desired number of MPI processes (see Section 2.3) even though the simulation runs only on one MPI process, which is MPI rank 0.

#### 2.2.1. Build phase

As MPI processes operate independently of each other during the build phase (see Figure 1), MPI rank 0 does exactly the same in a dry-run simulation as in the corresponding real simulation. The implementation of the dry-run mode for the build phase did not require any changes to code except for making it possible to set the NEST-kernel parameter num_processes at the beginning of a simulation.

As neurons are distributed in a round-robin fashion according to their GIDs, MPI rank 0 creates the neurons whose GIDs modulo the total number of MPI processes equals 0. Hence, the rank does not require any information from the fake ranks but it just needs to know the number of MPI processes, which is provided by the parameter num_processes.

Connecting two neurons also does not require any interaction with the fake ranks. As synapses are represented on the same MPI rank as their target neurons, rank 0 just needs to check whether the target neuron of a particular synapse is local in order to decide whether it should create the synapse. In real simulations, NEST uses MPI_Allgather to communicate spikes such that the spikes of each neuron will reach every MPI rank regardless of the existence of local targets. Therefore, the MPI rank of the source neuron does not need to be notified of a newly created connection.

As in real simulations rank 0 registers the rank-local neurons and synapses with the local neuron and connection infrastructure (see Figure 2).

#### 2.2.2. Simulation phase

While the implementation of the dry-run mode for the build phase required virtually no changes to the NEST code base, the extension of the dry-run principle to the simulation phase needs to address the problem that a major part of the neuronal network is missing. There are no remote neurons that send spikes to the dry-run process in every simulation cycle.

Therefore, NEST omits the MPI communication of the gather step in dry-run simulations; there is only one process running anyway. Instead, rank 0 fills the global spike buffer (see Figure 3C) with fake spikes. In real simulations, the buffer holds the GIDs of all neurons that spiked in the previous simulation cycle (see Section 2.1). In dry-run simulations, rank 0 generates the GIDs at random but it needs to comply with the structure of the global spike buffer in order to create a similar instruction flow as in a real simulation. As every rank has its own section in the global spike buffer and every thread has its own subsection, the dry-run process needs to make sure that every part of the buffer contains only the GIDs of neurons that are local on the corresponding rank and thread. We can assume a round-robin distribution of neurons such that gid % num_processes defines the rank and gid % (num_processes ^*^ local_num_threads) / num_processes defines the thread, where local_num_threads is the NEST-kernel parameter for the number of threads that run on each MPI process.

During the gather step of both dry runs and real runs each MPI rank collocates the MPI send buffer based on the spike-register entries (see Figures 3A,B, respectively). After this, in real runs the MPI communication of the buffers is triggered, whereas in dry-run mode each rank fills the global spike buffer with fake spikes using multiple threads.

It is not possible to obtain estimates of communication times from dry-run simulations. However, the NEST kernel variable send_buffer_size provides a good approximation for the send-buffer size of the corresponding real run. Based on this value realistic estimates of communication times can be obtained from MPI communication benchmarks. Generally, the communication time in real runs is very short compared to the overall runtime of the simulation phase (percentages in the lower single-digit range as shown in Section 3.1.3) so that the dry-run mode covers most part of the simulation phase anyhow.

The local_spike_counter is another kernel variable that is especially useful for benchmarking purposes. The variable keeps track of the number of spikes that the local neurons emit and hence enables the calculation of firing rates without using a spike detector.

We developed two versions of the dry-run mode: the static and the dynamic mode. The two modes differ in the way they determine the number of fake spikes in each simulation cycle.

##### 2.2.2.1. Static dry-run mode

The static mode does not aim on mimicking the dynamics of a real simulation, but it allows for direct control of the frequency of the spikes that enter the delivery step within every simulation cycle. This can be useful for very specific profiling needs. Therefore, in static mode the firing rate is a parameter controlled by the user. In each simulation cycle the entire global spike buffer including the part which belongs to rank 0 is filled with as many fake spikes as necessary to approximate the target firing rate. An exact match is hardly possible because the global spike buffer is at the lowest level subdivided in parts which correspond to a single h-step and thread, and because the number of spikes in such a part has to be an integer number. To obtain a close approximation even for low firing rates, the number of spikes per h-step and thread is varied between buffer parts such that the average firing rate is close to the target firing rate.

Please note that the firing rates of the neurons on MPI rank 0 are not directly affected by this procedure and can deviate from the target firing rate. However, as these spikes are ignored in further processing, this has negligible influence on the runtime of the simulation phase. One could argue that ignoring the spikes generated by rank 0 gives away important information. However, it belongs to the core concept of the static dry-run mode to have full external control over the number of incoming spikes. As we will see in the next section, the concept of preserving information is in contrast fully realized in the dynamic dry-run mode.

##### 2.2.2.2. Dynamic dry-run mode

The main objective of the dynamic dry-run mode is to mimic the spike dynamics of the corresponding real simulation as closely as possible on the single dry-run process. In simulated neuronal networks firing rates vary over time. In particular, neuronal populations with many recurrent connections show synchronization behavior, which means that the neurons of the same population tend to fire together and pause together. Thus, the number of spikes generated by a subset of the population is an indicator of the overall number of spikes generated by the entire population at a specific point in time. We use this as a principle for the dynamic dry-run mode: The number of fake spikes per fake rank is always equal to the number of spikes generated by the neurons on the dry-run process (MPI rank 0).

In dynamic dry-run mode the spikes of the neurons on rank 0 are not discarded but transferred to the global spike buffer. Hence, rank 0 needs to fill only the sections of the global spike buffer that belong to the fake ranks. To this end the MPI rank copies its own section of the buffer to the section of every fake rank; this may require increasing the buffer size first. Then every GID in the sections of the fake ranks is replaced with a randomly drawn GID that matches rank and thread of the currently processed part of the buffer (see Figure 3D). This is carried out using multiple threads.

### 2.3. User interface

In order to enable the dry-run mode, NEST users need to adapt their simulation scripts only marginally, which is demonstrated in the following PyNEST example. The simulation script creates a balanced random network (Brunel, 2000) of 100, 000 neurons with 10, 000 incoming synapses per neuron (80% excitatory, 20% inhibitory). Each neuron receives Poisson input of 5, 000 spikes per second.

**Table:** 

1	**import** nest
2	
3	nest . EnableDryrun ()
4	
5	nest . SetKernelStatus ({ ' num_processes ' : 24,
6	' dryrun_target_rate ' : 0.0})
7	
8	# *create neurons and devices*
9	nr = nest . Create ( ' iaf_neuron ', 100000)
10	pg = nest . Create ( ' poisson_generator ' ,
11	params = { ' rate ' : 5000.0})
12	sd = nest . Create ( ' spike_detector ' ,
13	params = { ' to_file ' : True })
14	
15	# *excitatory connections*
16	nest . Connect ( nr [:80000], nr ,
17	{ ' rule ' : ' fixed_indegree ' ,
18	' indegree ' : 8000},
19	{ ' weight ' : 40.0})
20	
21	# *inhibitory connections*
22	nest . Connect ( nr [80000:], nr ,
23	{ ' rule ' : ' fixed_indegree ' ,
24	' indegree ' : 2000},
25	{ ' weight ' : - 200.0})
26	
27	# *Poisson input to all neurons*
28	nest . Connect ( pg , nr ,
29	syn_spec ={ ' weight ' : 40.0})
30	
31	# *record spikes from all neurons*
32	nest . Connect ( nr , sd )
33	
34	# *simulate network for 10 s*
35	nest . Simulate (10000.0)

**Figure 4:**
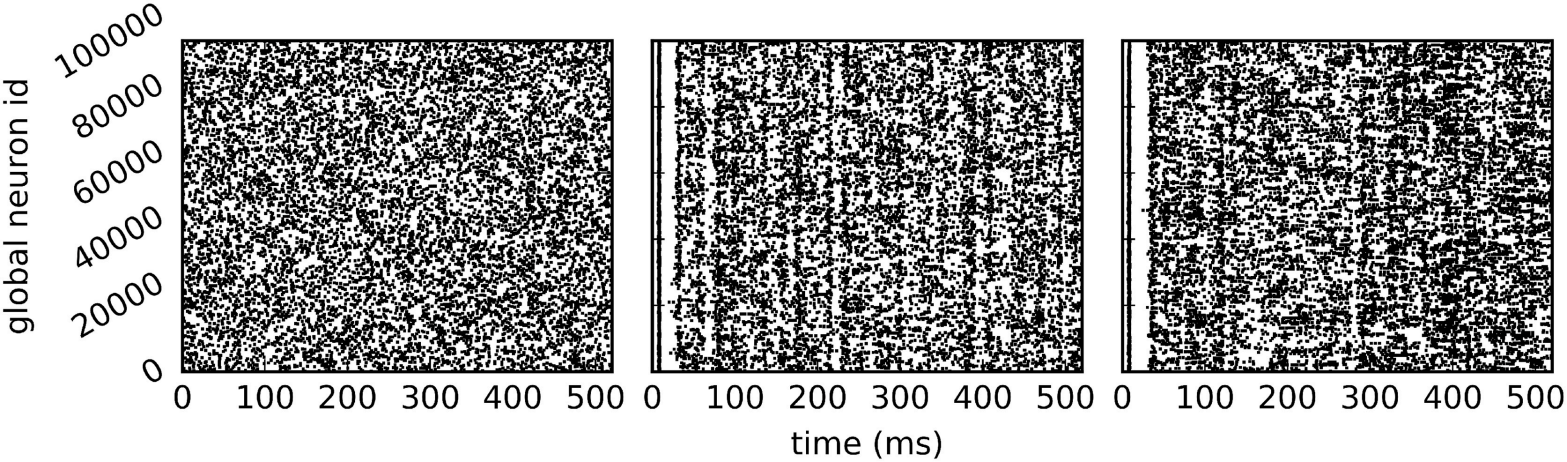
Spike output of the example script. Spikes of all neurons on rank 0 in the first 500 ms of a 10 s simulation **(A)** in static dry-run mode with a target firing rate of 5 Hz (average firing rate of 4.97 Hz), **(B)** in dynamic dry-run mode (average firing rate of 4.82 Hz), and **(C)** in the corresponding real run (average firing rate of 5.91 Hz) using 24 MPI processes.

The call to EnableDryrun at the beginning of the script initiates the dry-run mode and unlocks the dry-run parameters in the kernel dictionary. If the dry-run mode is enabled, the user can set the NEST-kernel parameter num_processes, which is otherwise not possible. In the above example num_processes is set to 24 such that the dry-run process behaves as if it was part of a simulation with 24 MPI processes. The parameter dryrun_target_rate defines the target firing rate of fake neurons in static mode. Any value greater than 0 enables the static mode whereas setting dryrun_target_rate to 0 enables the dynamic mode. Figure 4 shows the spikes of the neurons on MPI rank 0 in the first 500 ms of the simulation for the two dry-run versions and for the corresponding real run.

The dry-run mode can only be enabled in simulations that use one process (i.e., num_processes is initialized to one). Once it is enabled, the dry-run mode can only be disabled by a reset. Currently the method is not compatible with precise spike times (Hanuschkin et al., 2010; Morrison et al., 2007b), gap junctions (Hahne et al., 2015), or structural plasticity (Diaz-Pier et al., 2016); see Section 4.2.

The PyNEST command GetKernelStatus allows NEST users to inspect all kernel parameters such as num_processes. All dry-run parameters are tagged by a dryrun_ prefix. The kernel status dictionary also contains the local_spike_counter and the send_buffer_size.

## 3. Results

### 3.1. Validation of dry-run mode

In this section, we address the question if performance measurements (memory footprint, timings) that are carried out in dry-run mode are close to the values observed in real runs. This is important for proving the usefulness of the dry-run mode. As the number of processed spikes has a strong impact on NEST performance (see Section 3.1.2), special emphasis is given to the comparison of spike frequencies and patterns in dynamic dry-run mode. The testbed for benchmarking is a balanced random network (Brunel, 2000) with plastic synapses (Morrison et al., 2007a), which consists of two populations of integrate-and-fire neurons (80% excitatory, 20% inhibitory), where all excitatory-excitatory connections exhibit STDP (Zhang et al., 1998; Markram et al., 1997) and all other connections are static. The network is driven by random spikes emitted by a Poisson generator whose output frequency is scaled via the parameter η. The size of a single integration step (h-step) is set to 0.1 ms, the min-delay interval amounts to 1.5 ms. It is discussed in Section 4.2 why balanced random networks were chosen for validation.

As described before, in static dry-run mode there are two spike frequencies: The symbol *F* denotes the frequency of fake spikes and is a control parameter of the static mode; the symbol *F*_real_ is used for the frequency of the spikes generated by the neurons on the dry-run process (MPI rank 0). In dynamic dry-run mode, *F* is chosen as *F* = *F*_real_ in every simulation cycle, therefore only the symbol *F* is used when discussing the dynamic mode. The same holds for real runs, where the distinction between *F* and *F*_real_ is meaningless. An overview of additional important NEST application parameters is given in Table 1.

**Table 1:** Relevant NEST application parameters.

**Symbol**	**Explanation**
*N*	Number of neurons in the overall network
*N*_*M*_	Number of neurons per MPI rank
*K*	Number of incoming connections per neuron
*K*_*S*_	Scaling factor for the computation of *K*: *K* = *K*_*S*_ · 11 250
*M*	Number of (fake) MPI ranks
*T*	Number of threads per MPI rank
*F*	Mean spike frequency over simulation period
η	Scaling factor for the random input which drives the network

#### 3.1.1. Dynamics of the balanced random network

**Figure 5:**
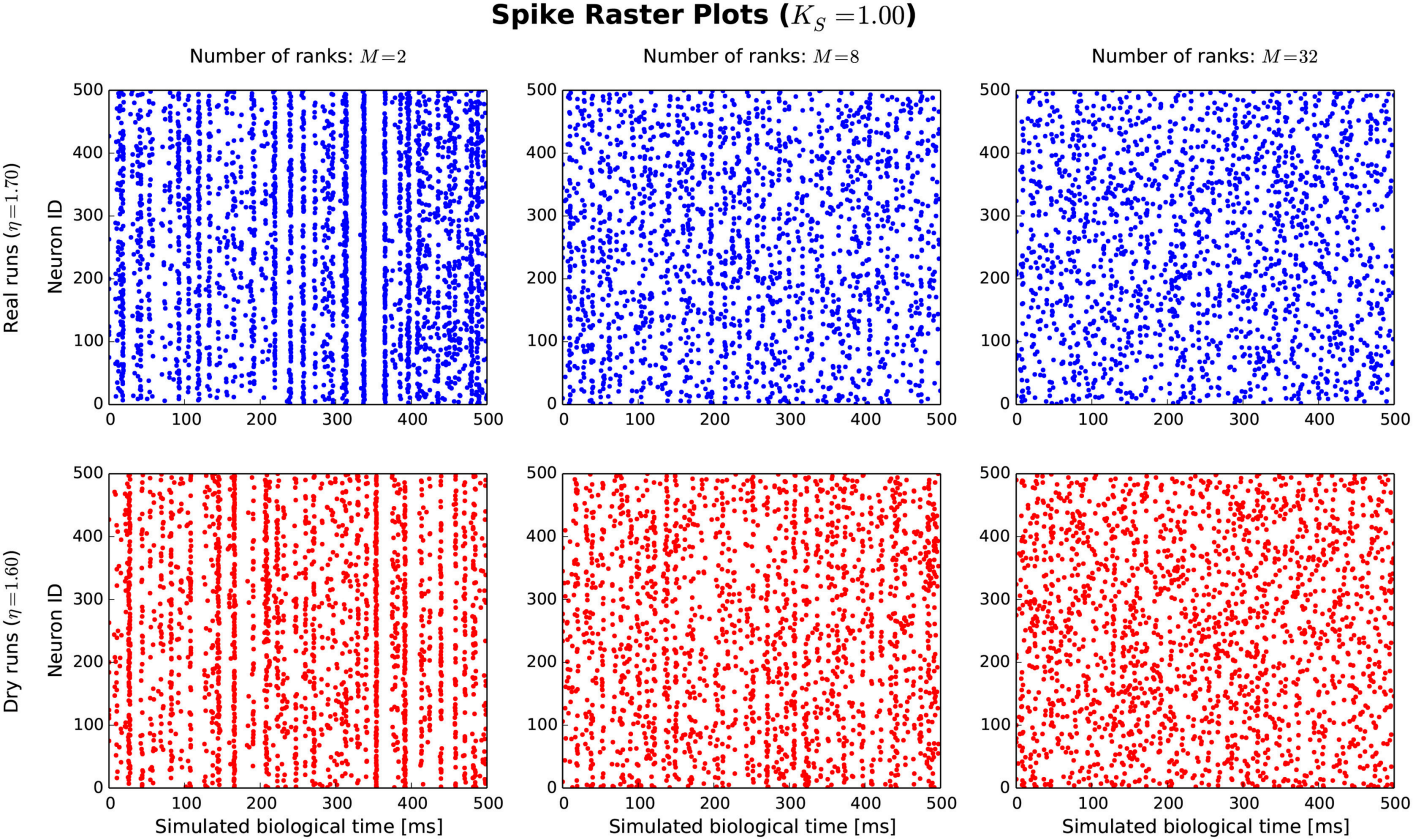
Spike raster plots for dynamic dry runs and real runs with different sizes of a balanced random network. Network size *N* is directly proportional to the number of ranks *M* (weak scaling design with approximately 5, 500 neurons per rank). The spikes of the first 500 neurons are shown over a simulated time of 500 ms. η values are slightly different for real and dry runs to enable better matching spike frequencies.

**Figure 6:**
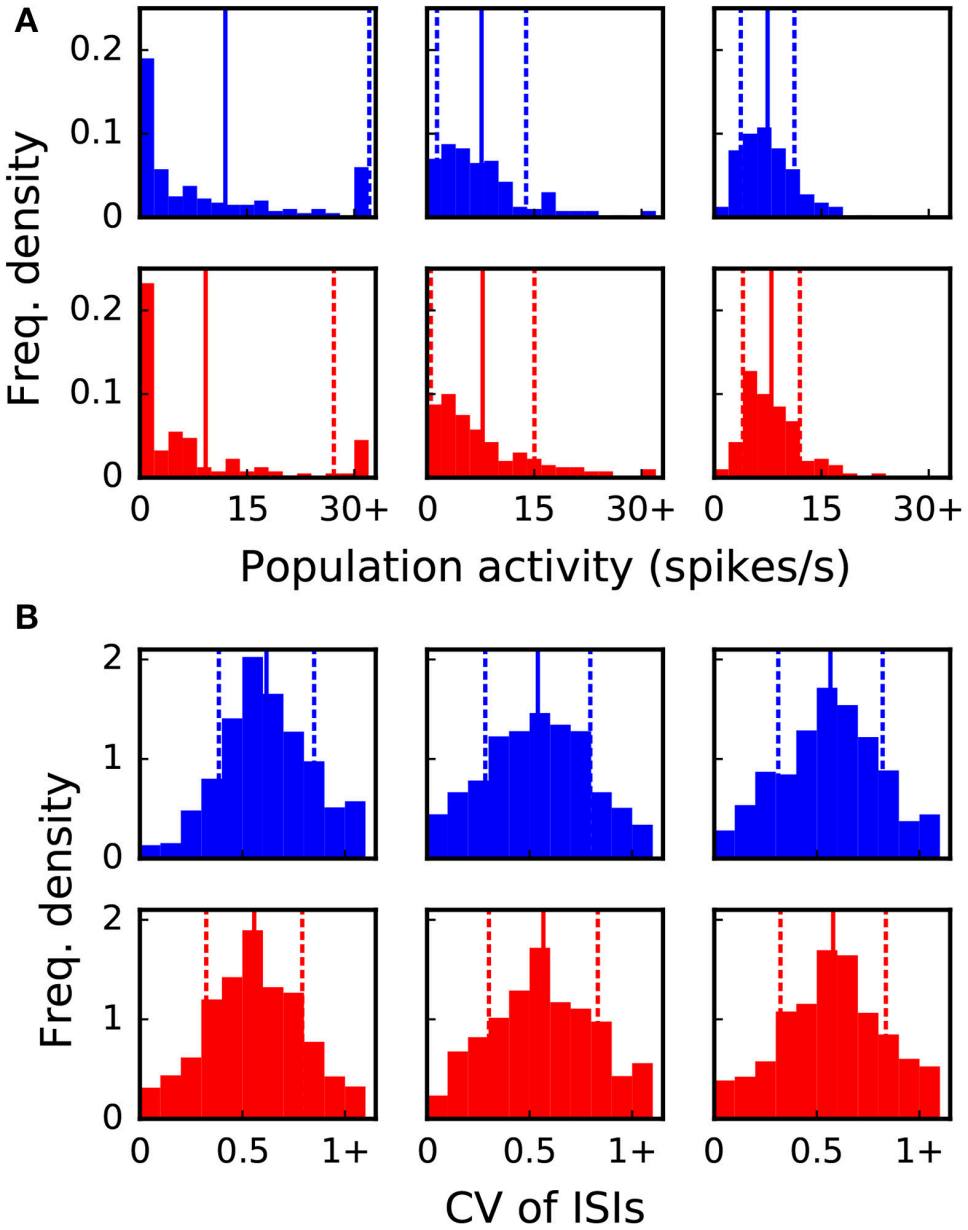
Statistical properties of the spike data shown in Figure 5. The six panels in **(A,B)** correspond to the six panels in Figure 5. Vertical lines and dashed lines indicate mean and standard deviation relative to mean, respectively. **(A)** Distribution of population activity. For successive time intervals of 2.5 ms the spikes of the recorded neurons were taken into account in order to determine the instantaneous population activity. Mean values are 12.0, 7.6, 7.5, 9.2, 7.8, 8.0 and standard deviations are 20.1, 6.2, 3.8, 17.9, 7.2, 4.0 spikes per second from left to right and top to bottom panel. **(B)** Distribution of coefficient of variation (CV) of inter-spike intervals (ISIs). The CV of ISIs was calculated for every neuron of the recorded population that spiked at least three times in the 500 ms interval; a percentage of 97, 77, 76, 89, 78, 79% (left to right, top to bottom) of the neurons fulfilled this requirement. Mean values are 0.62, 0.54, 0.57, 0.56, 0.57, 0.58 and standard deviations are 0.23, 0.26, 0.25, 0.23, 0.27, 0.26 from left to right and top to bottom panel.

The goal of the dynamic dry-run mode is to generate spiking dynamics similar to real runs. As a first step in the validation process, spike raster plots are compared qualitatively for varying sizes of a balanced random network (strict weak scaling design: The number of ranks *M* is varied, the number of neurons per rank is set to *N*_*M*_ = 5 500; the number of neurons in the whole network amounts to *N* = *N*_*M*_*M*; the number of incoming connections per neuron is set to a constant value of *K* = *K*_*S*_ · 11 250, *K*_*S*_ = 1.0). The results are depicted in Figures 5, 6 for different values of *M*; the top row of the plots shows the real runs (blue), the bottom row the dry runs (red; spike times and statistics of fake spikes). For small simulations with *M* = 2, strong synchronization patterns can be observed in the raster plots in Figure 5, which are similar between real and dry runs. Moving up to *M* = 8, synchronization is much weaker but still existent—both for real and dry runs. At *M* = 32 synchronization is no longer visible. This observation is confirmed by the distributions of population activity in Figure 6A: For *M* = 2 the distribution is scattered with many intervals of high activity and with a peak at extremely low activity, whereas for *M* = 8 and *M* = 32 the main body of the distribution is centered around 7.5–8.0 spikes per second. The distributions of coefficient of variation of interspike intervals for the dry-run simulations are also in good agreement with the results of the real runs (see Figure 6B); however, due to the short recording period of 500 ms these histograms should be taken with a grain of salt. In summary, the spike patterns of real runs are very closely mimicked by dynamic dry runs over a large range of network sizes.

**Figure 7:**
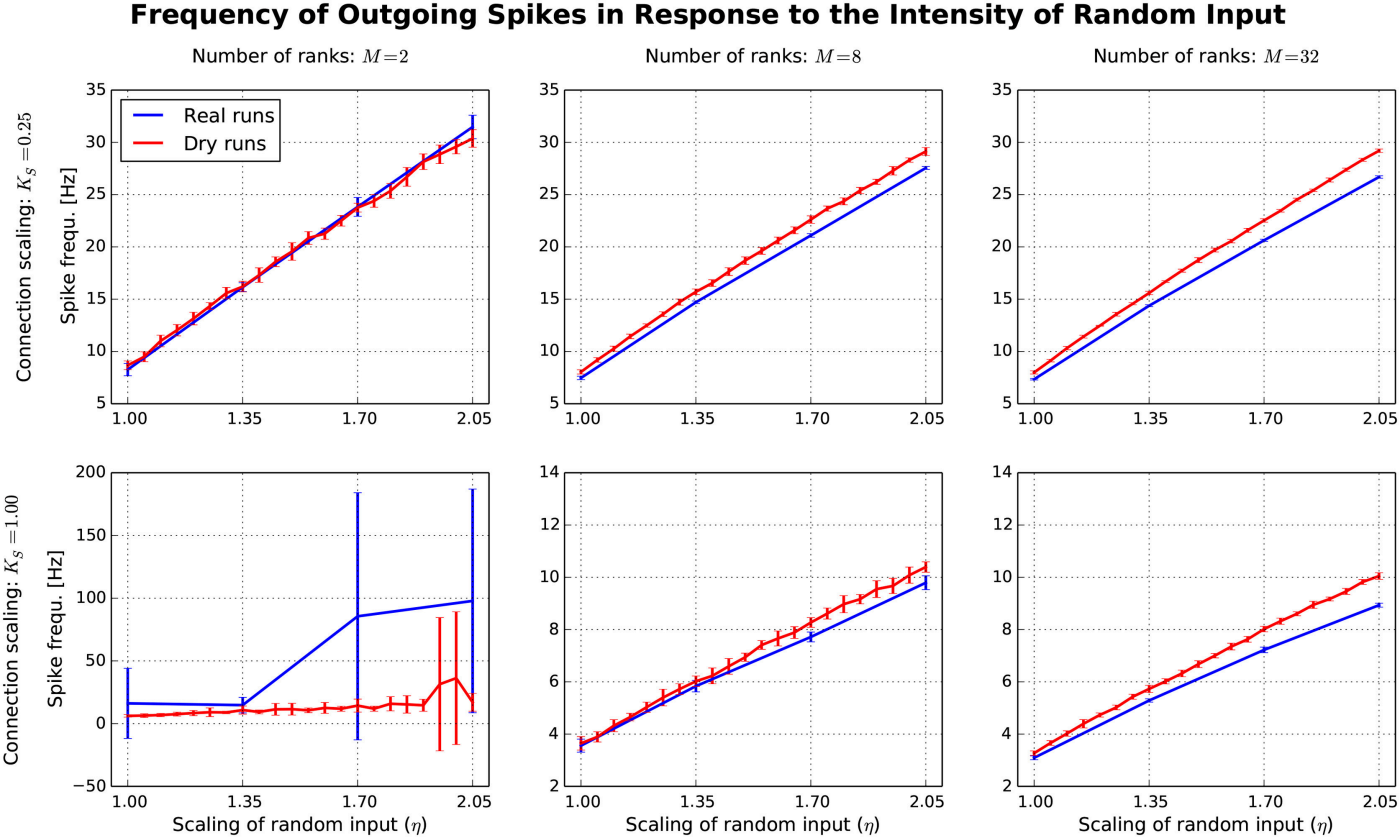
Each subplot shows how the spike frequency in a balanced random network depends on η (the parameter which scales the random input to the network), comparing dynamic dry runs (red) and real runs (blue). There were 10 repetitions with different random master seeds in each experimental condition, the error bars show the resulting standard deviations. Between subplots, *M* is varied as in Figure 5, and in addition the number of incoming synapses per neuron (according to the scaling factor *K*_*S*_; the number of incoming synapses per neuron amounts to *K* = *K*_*S*_ ∗ 11 250).

After having confirmed the qualitative match between real runs and dynamic dry runs, the next step is to compare spike frequencies systematically. In this experiment, the simulation size, the fan-in, and the parameter η are varied (*M* ∈ {2, 8, 32}; *K*_*S*_ ∈ {0.25, 1.0}; η ∈ {1.0, …, 2.05}). The dependent variable is the spike frequency *F* over 500 ms of simulated biological time (additional settings: *N*_*M*_ = 5 500, *N* = *N*_*M*_*M*, *K* = *K*_*S*_ · 11 250). In Figure 7, *F* is plotted against η for the different experimental conditions (real runs: blues curves; dry runs: red curves). For a small network with high connectivity (lower left subplot), the curve for the real runs is erratic with large variance. This is to be expected, since *N* = 11 000 neurons are simulated with *K* = 11, 250 synapses per neuron, resulting in strong recurrence and highly unstable dynamics. This specific behavior is not well captured by the dry-run results. However, this is a minor shortcoming, because the real simulation has only *M* = 2 ranks, which is not a typical use case for the dry-run mode. As soon as we reduce the connectivity (upper left subplot), real and dry runs match very well over the whole η range with *F* being an approximately linear function of η. Increasing the number of ranks (subplots in the middle and right column of Figure 7) illustrates that *F* barely depends on network size, but strongly on network connectivity—the spike frequencies are much larger for *K*_*S*_ = 0.25 compared to *K*_*S*_ = 1.0. In addition, it becomes visible that the dry runs overestimate the spike frequency of the real runs systematically. However, the gap is not large and can easily be reduced by choosing a smaller η value for the dry runs. For the balanced random network model, a reduction of η between 5 and 10% can be derived from these curves as a reasonable rule of thumb to arrive at dry-run spike frequencies which are close enough to real runs to be perfectly usable for profiling and related purposes.

In contrast to the dynamic dry-run mode, the static dry-run mode produces always a homogeneous pattern of incoming spikes without any synchronization patterns. This is clearly visible in Figure 4 and a direct consequence of the underlying mechanism of fake spike generation (see Section 2.2.2).

#### 3.1.2. Runtimes and memory consumption

So far, we have looked at spike frequencies in the dynamic dry-run mode and at rather small simulation sizes. In this subsection, runtimes and memory consumption are included in the comparison, both for the static and the dynamic version of the dry-run mode. Furthermore, a wide range of simulation sizes is covered. Data was collected on the supercomputer JUQUEEN^1^ at Forschungszentrum Jülich. NEST was used in revision 10694 from the codebase in the main NEST SVN repository^2^, supplemented by the code for dry-run functionality at revision 11501.

In a factorial experiment, the number of ranks *M* was varied between 32 and 28672 (*M* ∈ {32, 128, 512, 2048, 4096, 8192, 16384, 28672})^3^, the number of threads *T* per rank between 4 and 64 (*T* ∈ {4, 8, 16, 32, 64}), and the number of neurons per rank between a half-fill and a full-fill setting. At the latter setting, nearly the whole available main memory in each compute node was consumed by NEST data structures, in the former setting only half of it. Due to a limited amount of available computing time, only 71 of the 80 experimental conditions of the full factorial design were included in the experiment. In each condition, a real run, a static dry run, and two dynamic dry runs were executed. The η value in the real runs was set to η = 1.685, resulting in a spike frequency of approximately 7 Hz. Therefore, *F* was also set to *F* = 7 Hz in the static dry runs. The two dynamic dry runs differed with regard to the parameter η. One was carried out with η = 1.685 like in the real runs, one with η = 1.56 to match the spike frequency *F* of the real runs (according to the rule of thumb suggested in Section 3.1.1).

**Figure 8:**
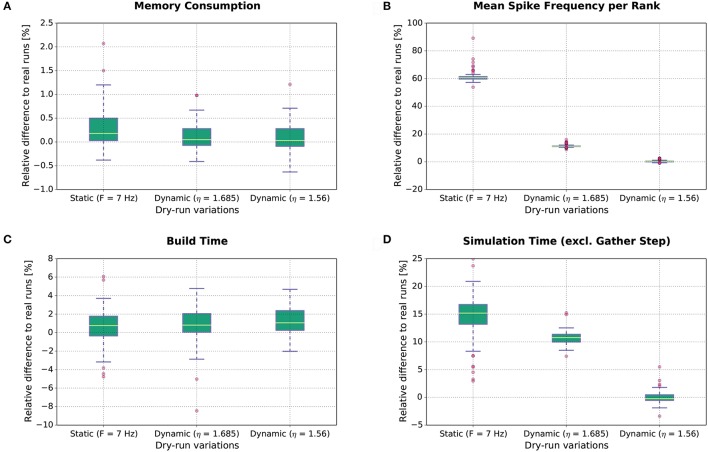
Dry-run performance relative to corresponding real simulations. The relative difference between real and dry runs is computed by (*d*_dry_ − *d*_real_)/*d*_real_ with *d* being the measured value.

The results of this study are shown in Figure 8. Each boxplot depicts the distribution of the relative differences between the real runs and the dry runs over all experimental conditions for the different variations of the dry-run mode (left: static; middle: dynamic with η = 1.685; right: dynamic with η = 1.56) and for different measures (A: Memory consumption; B: Mean real spike frequency per rank *F*_real_; C: Build time; D: Simulation time excl. gather step). Boxplot B reveals that the mean spike frequency *F*_real_ is very well matched between real runs and the dynamic dry-run mode with η = 1.56. For η = 1.685, the median of the relative differences in spike frequencies amounts to approximately 12%, and for the static dry-run mode to approximately 60% (although *F* = 7 Hz like in the real runs; thus, in static mode we observe *F* ≠ *F*_real_).

The differences in spike frequency have a direct impact on simulation times (measurements exclude the gather step which is considered separately in Section 3.1.3). For the dynamic dry-run mode with η = 1.56, the median of the relative differences to the real runs is close to 0%; deviations are mostly within the range [−5%;5%]. This shows clearly that the dynamic dry-run mode with adjusted η value can faithfully generate time measurements which are representative for real large-scale simulations. Even with a non-adjusted η value (η = 1.685), the median of the relative differences amounts only to approximately 11% with rather small variability. However, static dry runs take about 15% more simulation time^4^. This is combined with rather high variability in the range [3%;25%]. Thus, the static dry-run mode is not as well suited as the dynamic dry-run mode for the estimation of the overall simulation time of real runs.

Regarding memory consumption (boxplot A), the dynamic dry-run mode comes very close to real simulations. Both for η = 1.56 and η = 1.685 the median of the relative differences amounts to approx. 0%; variability is approximately restricted to the range [−0.7%;1.2%]. In static mode, differences are slightly larger with a maximum of over 2%. These results show that dry runs provide a realistic assessment of the memory consumption of real simulations.

The same holds for build times (boxplot C). In the build phase of NEST (creation of neurons and network wiring), exactly the same code path is executed in dry runs and real runs. Therefore, the expected runtime difference is 0%; the observed median of the relative runtime differences amounts to approximately 1% in all dry-run variations. The variability is mostly confined to the range [−5%;5%]. This seems to be the normal variation range on JUQUEEN when exactly the same code is executed at different points in time. Therefore, we conclude that the simulation time measurements with the dynamic dry-run mode and adjusted η are already as close to real runs as achievable in practice.

#### 3.1.3. Runtime of the gather step

**Figure 9:**
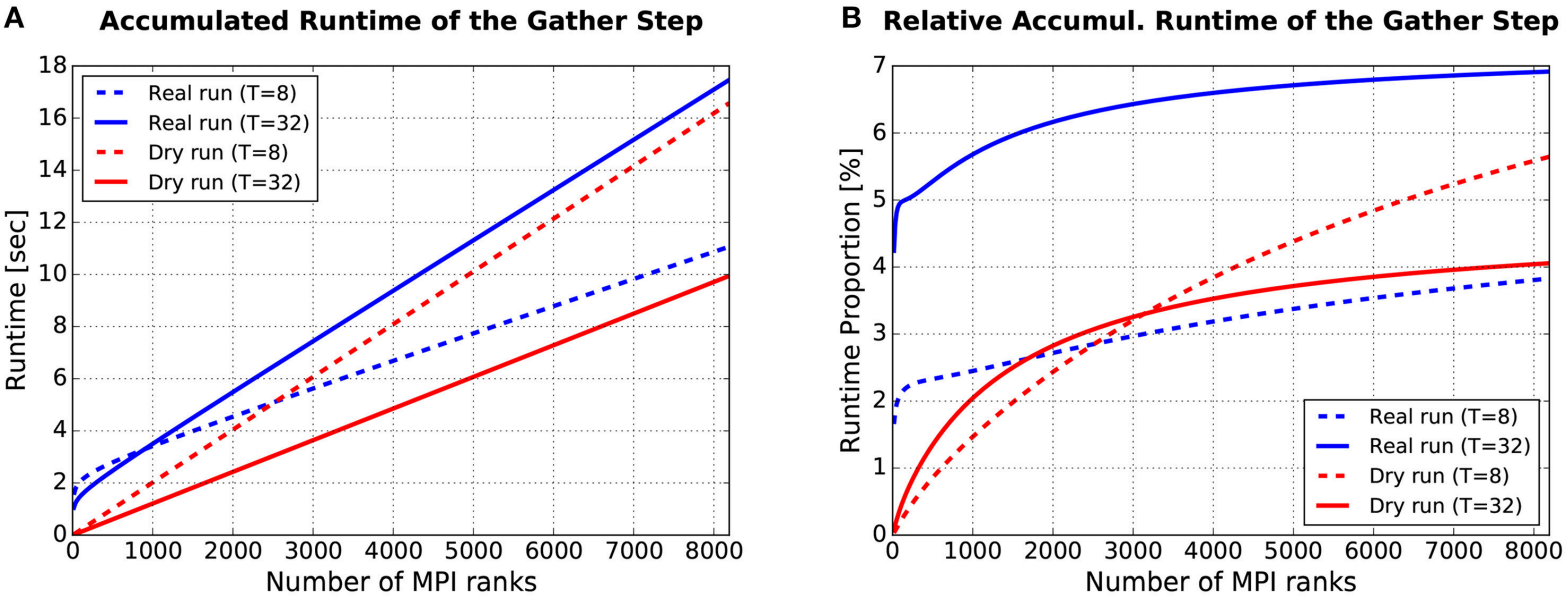
Runtime required for the gather step, accumulated over all simulation cycles. **(A)** Absolute values. **(B)** Relative values: Gather runtime as percentage of the overall simulation time (incl. deliver events, update nodes, and gather events). For more details see text.

From a single-compute-node perspective, the substantial difference between a dry run and a real run is what happens during the gather step in each simulation cycle: Fake spike generation in dry runs, MPI communication in real runs. For users of the dry-run mode, it would be unfavorable if fake spike generation required a lot more time than MPI communication. Figure 9 supports the claim that this is not the case, at least not for typical simulation settings and assumptions using JUQUEEN.

The plot in Figure 9A shows how the runtime of the gather step (accumulated over all simulation cycles) depends on the number of (fake/real) MPI ranks *M*. Four curves are depicted, the blue ones for real runs, the red ones for dynamic dry runs. The dotted lines are for *T* = 8 threads per rank, the solid ones for *T* = 32 threads per rank. These curves were generated with semi-empirical performance models (Hoefler et al., 2011) of the runtime of the gather step (a separate model for dry runs and real runs). These models depend in turn on model-based estimations of the send buffer size. The models were fitted to a subset of the data from the experiments described in Section 3.1.2 (real runs with η = 1.685 and dry runs with η = 1.56, resulting in approximately the same mean spike frequency of *F* = 7 Hz; *T* ≤ 32). The model fit was very good with the coefficients of determination being in the range between *R*^2^ = 97.9% and *R*^2^ = 99.7%. Thus, the shown curves are representative for experimental data collected on JUQUEEN. It can be observed that the dry-run mode is faster for *T* = 32, and will continue to be so even beyond the shown limit of *M* = 8192 ranks. In contrast, for *T* = 8 real runs are faster if the number of ranks is larger than *M* ≈ 2, 500.

As supplementary information, Figure 9B shows the runtime of the gather step in relation to the overall runtime of the whole simulation phase. To estimate the runtime of the whole simulation phase, an additional performance model for the steps “deliver events” and “update nodes” within the simulation phase is required. Such a model was presented in Schenck et al. (2014) and is re-used here in slightly modified form, resulting in a model fit of *R*^2^ > 99.8% for both real and dry runs. The percentages in Figure 9B are in the single-digit range. The largest values can be observed for real runs with *M* = 8192/*T* = 32: For this setting, the runtime of the gather step converges to about 7% of the overall simulation time.

The data proves in summary that the runtime of the gather step is in the same order of magnitude for real runs and dynamic dry runs, and that the generation of fake spikes does not cause any systematic and considerable performance penalty. Furthermore, the gather step contributes only by a small part to the overall simulation time and can therefore be ignored in dry-run measurements without the fear to miss an important piece of information.

### 3.2. Use cases

As outlined in the introduction, the development of the dry-run mode was motivated by several use cases. Generally speaking, the dry-run mode allows to estimate the memory footprint and the runtime of a NEST simulation—the former very precisely, because the memory footprint depends mainly on the number and type of neurons and connections and the respective infrastructure which is created in exactly the same way during the build phase of real runs and dry runs—the latter with good accuracy as described in Section 3.1.2.

From the perspective of a NEST user, these capabilities of the dry-run mode are useful in the areas of cluster computing and supercomputing for specifying simulation parameters in advance without the need for test runs on the full machine. As NEST simulations on supercomputers are more limited by the available memory than they are compute-bound, the challenge for a NEST user is to squeeze as many nodes and connections on a single node as possible. Although a memory model for NEST exists (Kunkel et al., 2012), adjustments are necessary in practice to get the absolute maximum. To reach this maximum, the network size has to be increased in an iterative way over several NEST runs, but these runs can be dry runs, which saves a large amount of core hours and effort.

A similar argument holds for the following use cases which rely on estimating the runtime in advance. It is common practice at computing centers that users only have a limited amount of computing time at their disposal, and that it is necessary to specify in advance how long a compute job will run on a system at maximum. Very often, the maximum runtime can only be guessed if new scaling sizes or simulation parameter settings are explored. However, with the help of the dry-run mode it is possible to get a very good estimate in advance without the need for full-scale test runs. This is a considerable advantage from the user perspective and helps to manage the granted computing time in a better way. In addition to this, measurements that are carried out with the dry-run mode can be used to collect data for scaling plots when writing computing time applications.

So far, use cases from the user perspective were considered. However, estimating the memory footprint and the runtime is also a highly valuable tool for NEST developers. The collection of profiling data is carried out rarely at large scale because of the associated computational costs and effort—although it is common wisdom in the HPC community that the behavior of parallel codes depends strongly on simulation size and the number of compute nodes involved. Some optimizations may be very useful at small scale but cause a performance bottleneck at large scale. The dry-run mode enables the developers to investigate the behavior of their code at different scales at low cost. In the following, two specific applications of the dry-run mode from the developer perspective are presented.

#### 3.2.1. Identifying inefficiencies at large-scale

Many performance problems in HPC applications only show up in large-scale simulations because the resources wasted by minor inefficiencies grow disproportionately high with simulation size. Thus, they are barely noticeable when running on a small number of MPI ranks, but take up a considerable amount of computing time when running full scale on a supercomputer. Unfortunately, profiling of large simulation runs is rarely done because of the accompanying costs in terms of core hours and queueing time. In this respect, the dry-run mode is of great help because it allows the creation of performance profiles for large-scale simulations on a single rank (excl. MPI communication). Hence, the dry-run mode facilitates systematic profiling as it shortens turn-around times and requires fewer data to be processed. This shortens the development cycles for scalable NEST code.

**Figure 10:**
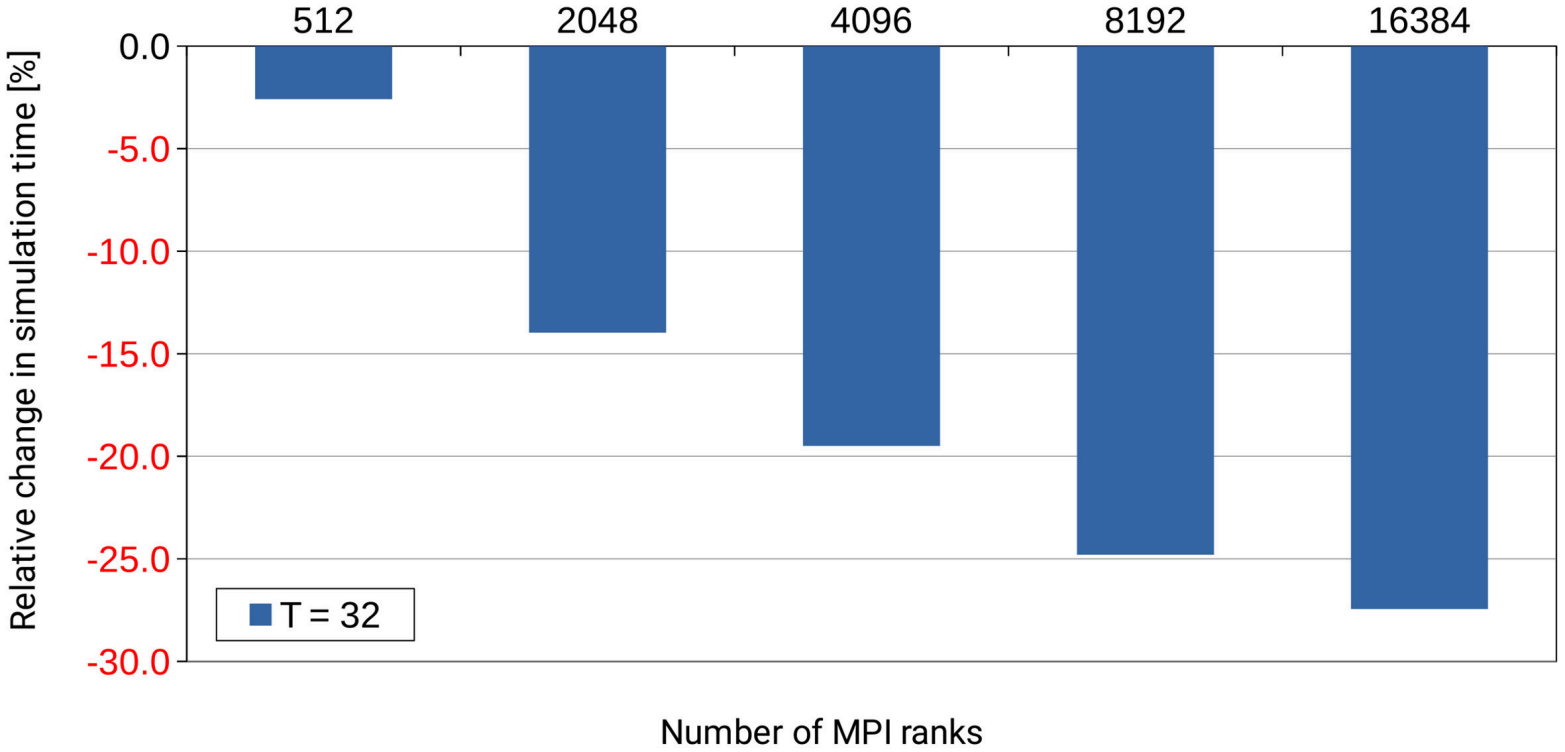
Performance gain for the simulation phase of NEST by changes to the Time class and to the handling of Time objects in the NEST code base. *T* denotes the number of threads per MPI rank. The number of MPI ranks is varied along the x-axis. The percental changes are reported relative to the original code version. Performance data was collected on the supercomputer JUQUEEN with a pre-release version of NEST 2.4.0, combined with an early implementation of the dry-run mode and (conceptually) one compute node per MPI rank. A balanced random network was used as test bed in a close to maximum memory filling setting.

Already during development of the dry-run mode we identified an inefficiency in the NEST code base that occurs only at large scale. NEST uses a Time class to store time stamps as Time objects. These objects allow simple conversion between different units of time. In small-scale runs up to 32 MPI ranks, the amount of computing time spent in the Time class was completely negligible and never considered a problem. However, when inspecting profiles and trace data^5^ which was created with a dry run of a large simulation with several thousands of (fake) MPI ranks, it turned out that operations on Time objects used up a considerable amount of the computing time during the simulation phase of NEST. By fixing this inefficiency through changes to the Time class itself and by improving the handling of these objects^6^, the required computation time was reduced by more than 25% as shown in Figure 10. This example demonstrates the usefulness of the dry-run mode for the development of highly scalable code.

#### 3.2.2. Performance modeling

The goal of performance modeling is to develop quantitative models which predict the runtime or other performance characteristics of an application depending on simulation parameters and scaling size. Performance models allow predictions for any simulation size, enable predictions of the consequences of algorithmic changes, and can guide code development for a large parameter and scaling range. Robust models with good generalization ability result from the semi-empirical approach in which the model itself is based on algorithmic complexities which are derived from source code or become effective during runtime (Hoefler et al., 2011). Free model parameters are determined by fitting the model to experimental data, i.e., measurements obtained from simulation runs. Such a model was developed in Schenck et al. (2014) for the runtime of the simulation phase of NEST, based on experimental data from JUQUEEN for a scaling size of up to 16,384 compute nodes. Because at that time, in 2014, the dry-run mode for the simulation phase did not yet exist, data collection for the performance model was very expensive in terms of required core-hours.

With the help of the dry-run mode as it is available now, data collection for performance modeling is rather effortless and cheap in comparison. Recently, a performance model for the runtime of the simulation phase of NEST was created which considerably extends the work by Schenck et al. (2014). In a full experimental design, comprising six factors, one of them being code variations of NEST, data for more than 5000 experimental conditions were collected on JUQUEEN, even going beyond the actually available maximum scaling size. Valuable insights could be gained from the resulting performance model about future code development directions^7^. Without the dry-run mode, such an endeavor would not have been feasible.

## 4. Discussion

### 4.1. General remarks

We presented the dry-run mode for NEST, by which it is possible to mimic the behavior of large-scale simulations on a single compute node. The results show that the generated data, such as spike patterns, memory consumption, and runtimes, is similar to corresponding real runs, at least for the dynamic version of the dry-run mode. Furthermore, two use cases for developers were explained in detail: With the help of the dry-run mode, large amounts of data for profiling and performance modeling can be collected without the need to employ more than one compute node for each data point. This saves huge amounts of core hours on large clusters or supercomputers and shortens development cycles. Furthermore, new algorithms can be tried out without wasting precious supercomputer resources, and the suite of automated software tests for NEST (Eppler et al., 2009b) can be extended to cover also large-scale simulations.

In a similar vein, users of NEST can use the dry-run mode to estimate in advance the required memory consumption and runtime of large-scale simulations. This helps to make better use of the available computing resources by saving core hours on test runs, by running full simulations with optimal parameter settings, and by enabling users to generate scaling data for compute-time proposals in an inexpensive way.

It is important to note that the same cannot be achieved by just running small simulations instead of large simulations. The connection infrastructure generated during the build phase of NEST differs considerably depending on simulation size, partly qualitatively, and of course quantitatively. The subsequent simulation phase uses these data structures for processing and shows therefore also different runtime behavior depending on overall simulation size.

### 4.2. Restrictions of the dry-run mode

The dry-run mode in its current form has two restrictions. The first restriction belongs inherently to the concept: the non-consideration of MPI communication and synchronization, i.e., in NEST terms the non-consideration of the gather step during the simulation phase. This restriction is not severe as we have shown in the results section. Generally, the gather step is very short in real runs. In the data from the supercomputer JUQUEEN reported in Section 3.1.2, it consumes between 1 and 8% of the overall simulation runtime. In case it is desired to estimate the time required for the gather step as well in advance, the dry-run mode helps at least insofar as it also predicts the size of the send buffers used for MPI operations. In combination with benchmarks of the MPI_Allgather operation on the respective cluster, the time required for MPI communication can be calculated.

The second restriction concerns the current focus on balanced random networks where the inhibitory and the excitatory subpopulation show the same firing rates and spike characteristics (especially the same synchronization patterns). Such network models are good-natured because their overall spike patterns are invariant with regard to network size if the neurons are sparsely connected (Brunel, 2000). These are important reasons why dynamic dry runs exhibit very similar spike patterns and frequencies as the corresponding real runs. For NEST developers and their typical use cases the confinement to balanced random networks is unproblematic because these networks can be parameterized in various ways to cover most base scenarios relevant for profiling and performance modeling (e.g., low vs. high connectivity, low vs. high spike rate, or with vs. without plastic synapses). However, NEST users may want to simulate networks with rich internal structure, consisting of many different subpopulations with varying characteristics, resulting in complex spike patterns. For such networks it is less clear whether a dry run will yield a simulation runtime similar to a real run. In any case, operations during the build phase are exactly the same between dry and real runs, and since nearly all of the required memory is allocated during network wiring, at least the estimation of the memory usage and of the runtime of the build phase will be quite accurate even for complex networks.

To get reliable estimates of simulation runtimes even for complex structured networks via the dynamic dry-run mode, it will be necessary to identify each subpopulation in the network and to extrapolate the spiking activity of each subpopulation on the single existing rank to the whole network. This is a topic of ongoing research. Furthermore, it is planned to investigate how the dry-run mode could be extended to cover very recent and advanced NEST features like structural plasticity (Diaz-Pier et al., 2016) and gap junctions (Hahne et al., 2015), which require MPI communication outside the standard spike-communication framework.

### 4.3. Applicability of the dry-run concept

In conclusion, the dry-run mode is an important contribution to the software-development framework of NEST. It facilitates the maintenance and further development of NEST as an open source project with a general-purpose orientation and extreme scalability. Furthermore, the basic idea of the (dynamic) dry-run mode is applicable to other parallel applications in a straightforward way if the following preconditions are fulfilled:

- The buildup of basic data structures on each rank does not depend on actions on other ranks.
- Statistical properties of the data that is generated during the simulation can be inferred from the single existing rank in dry-run mode. Especially, this needs to be true for those properties which are relevant for the simulation runtime, and which are employed to generate fake data in place of real data.
- The replacement of real data by fake data does not change the overall simulation dynamics in a way that considerably affects simulation runtimes or memory consumption.

These preconditions hold in principle for parallel implementations of Monte Carlo methods in which each compute node carries out an equally sized set of random experiments which is large enough to be representative for the whole simulated sample, or in which the impact of random events is computed in parallel (e.g., Carvalho et al., 2000). Furthermore, simulation algorithms based on spatial decomposition are candidates for a dry-run mode, at least if work is distributed equally over all compute nodes and stays (nearly) constant throughout the simulation (e.g., during the simulation of the dynamics of homogeneously distributed molecules with software like “ls1 mardyn”; Niethammer et al., 2014). And last but not least, simulators used in neuroscience like NEURON (Carnevale and Hines, 2006; Migliore et al., 2006) or NCS (“NeoCortical Simulator”) (Tanna, 2014) could profit from a dry-run mode similar to the one in NEST because the basic challenges are very similar (i.e., neurons distributed over MPI ranks, spike exchange via MPI communication).

## Author contributions

All authors listed, have made substantial, direct and intellectual contribution to the work, and approved it for publication.

### Conflict of interest statement

The authors declare that the research was conducted in the absence of any commercial or financial relationships that could be construed as a potential conflict of interest.

## Footnotes

^1^Each compute node of JUQUEEN is equipped with a 16-core PowerPC-A2 CPU and 4-fold SMT (64 hardware threads) and 16 GB of RAM. The max. number of usable compute nodes amounts to 28, 672.

^2^URL: https://trac.nest-initiative.org.

^3^The setting *M* = 28, 672 corresponds to using full JUQUEEN; each compute node of JUQUEEN hosted exactly one MPI rank in all experiments.

^4^The explanation for this increase in simulation time is as follows: In static dry-run mode, the dynamics of the neurons on the single existing rank are quite different to the real simulation. They produce actually more outgoing spikes than in a real simulation as shown in Figure 8B. This entails that more spikes need to be processed when updating STDP synapses; therefore the increase by 15% is observed.

^5^Generated with HPCToolKit (Adhianto et al., 2010) via statistical profiling.

^6^Credit for the code changes goes to Alex Peyser from the SimLab Neuroscience at Forschungszentrum Jülich and to WS (Peyser and Schenck, 2015).

^7^A publication about this study is in preparation.
